# The effect of tranexamic acid on artificial joint materials: a biomechanical study (the bioTRANX study)

**DOI:** 10.1007/s10195-014-0312-0

**Published:** 2014-08-05

**Authors:** Sattar Alshryda, James M. Mason, Praveen Sarda, T. Lou, Martin Stanley, Junjie Wu, Anthony Unsworth

**Affiliations:** 1 Departments of Trauma and Orthopaedics, Central Manchester University Hospitals, Oxford Road, Manchester, M13 9WL Lancashire UK; 2 Departments of Trauma and Orthopaedics, Medway Maritime Hospital, Windmill Road, Gillingham, Kent, ME7 5NY UK; 3 School of Medicine and Health, Wolfson Research Centre, Queen Campus, Durham University, Stockton on Tees, TS17 6BH UK; 4 Centre for Biomedical Engineering School of Engineering and Computing Sciences, Durham University, Durham, DH1 3LE UK

**Keywords:** Topical, Local, Tranexamic acid, Arthroplasty, Joint replacement, Blood loss, Blood transfusion, Wear rate, Surface topography, Biomechanical profile

## Abstract

**Background:**

Tranexamic acid (TXA) has been successfully used to reduce bleeding in joint replacement. Recently local TXA has been advocated to reduce blood loss in total knee or hip replacement; however, this raised concerns about potential adverse effects of TXA upon the artificial joint replacement.

**Materials and methods:**

In this biomechanical study we compared the effects of TXA and saline upon the following biomechanical properties of artificial joint materials—(1) tensile properties (ultimate strength, stiffness and Young’s modulus), (2) the wear rate using a multi-directional pin-on-plate machine, and (3) the surface topography of pins and plates before and after wear rate testing.

**Results:**

There were no significant differences in tensile strength, wear rates or surface topography of either ultra-high-molecular-weight polyethylene pins or cobalt chromium molybdenum metal plates between specimens soaked in TXA and specimens soaked in saline.

**Conclusion:**

Biomechanical testing shows that there are no biomechanical adverse affects on the properties of common artificial joint materials from using topical TXA.

**Level of evidence:**

V

## Introduction

A major cause of artificial joint failure is the loosening that occurs as a consequence of wear and tear of the bearing surfaces. Even a well performing prosthetic joint releases billions of microscopic wear particles (debris) into the joint space. When an excessive amount of debris is generated it may stimulate a severe body reaction in the capsular tissues and bone leading to inflammation and osteolysis. The resulting loss of supporting bone may lead to loosening of the implants requiring difficult revision surgery.

Most artificial joints consist of two metal surfaces, commonly made of cobalt chromium molybdenum (Co–Cr–Mo) alloy and an insert, commonly made of ultra-high-molecular-weight polyethylene (UHMWPE). The mechanical properties of these two materials are influenced by several chemical and physical factors. Oxidation breaks down the molecular chains in UHMWPE resulting in increased brittleness and reduced resistance to crack propagation. Historically, there have been unfortunate consequences within artificial joints when unexpected and unwanted chemical or physical reactions led to severe wear and joint failure. One example arose from the storage of artificial implants in air-filled packets after sterilisation using gamma radiation. This mode of storage led to gradual oxidation of the UHMWPE and deterioration of its mechanical properties. This was partly overcome by storage within a vacuum-sealed packet. Another example was post-irradiation thermal treatment to reduce free radicals in the polyethylene. This could cause a partial reduction in crystallinity of the material that, in turn, reduced the resistance to crack propagation [1].

Intravenous tranexamic acid (TXA) has been shown to reduce blood loss and transfusion in joint replacement [2–16]; however, fear of systemic side-effects precludes its wide use in orthopaedic practice. Locally administered TXA has been advocated as a better alternative due its easier preparation and administration, higher concentration at the bleeding site and less systemic side-effects [17–21]. There is a legitimate concern that TXA might adversely affect the biomechanical properties of artificial implants, especially the wear rate which might lead to subsequent loosening and failure. A rigorous search of the published literature, contact with manufacturers and contact with the community publishing on the use of TXA uncovered no research to address this issue. Consequently, this is the first study to investigate the effect of local TXA upon artificial joint biomechanical properties and performance. The research is named the bioTRANX study.

## Materials and methods

**Fig. 1:**
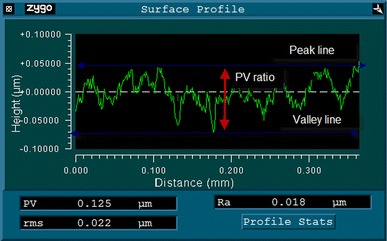
Peak-valley ratio (PV): the distance between the highest peak and the deepest valley over the entire evaluation length
  - Root mean square roughness (rms): the standard deviation of the surface roughness measurements relative to mean plane of all the data.
  - Arithmetic mean roughness (*R*_*a*_): the average roughness or deviation of all points from a plane (centre line) fitted to the test surface (Fig. 2).

In this biomechanical study we investigated the effects of TXA on the following biomechanical properties of artificial joint materials as surrogates for long-term effects:

1. Tensile properties of the UHMWPE (ultimate strength, stiffness and Young’s modulus).
2. The wear rate using a multi-directional pin-on-plate machine. Pins were made of UHMWPE and plates were made of Co–Cr–Mo.
3. The surface topography of pins and plates before and after wear rate testing:
  - Peak-valley ratio (PV): the distance between the highest peak and the deepest valley over the entire evaluation length (Fig. 1).

**Fig. 2:**
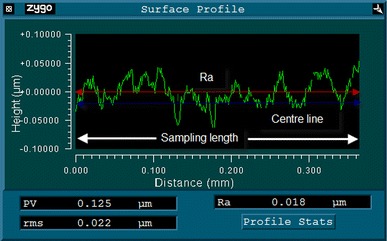
Surface roughness average (*R*_a_): the arithmetic mean roughness of all points from a plane (*centre line*) fitted to the test surface

All tested materials are made from identical materials used in the DePuy artificial knee joint replacement (P.F.C.^®^ Sigma^®^ Revision Knee System).

**Fig. 3:**
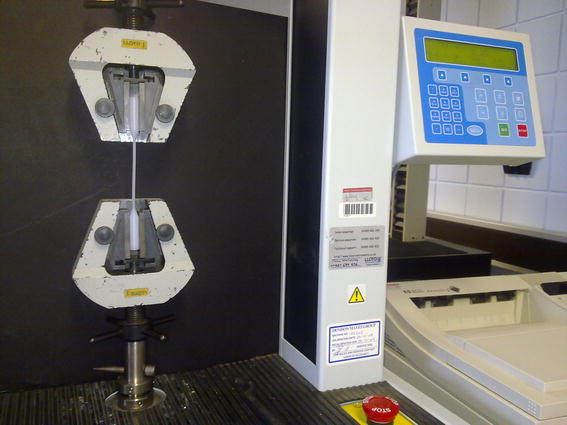
Tensile testing machine with a mounted specimen made of UHMWPE

*Tensile testing* was conducted by gripping the ends of a standardised test specimen made of UHMWPE in a tensile testing machine (Fig. 3) and then applying a gradually increasing axial load until failure occurred. Several calculations to describe the biomechanical properties of the material were obtained. Stress is the force applied per unit cross-sectional area (N/m^2^). Strain is the change in length divided by the original length. The stress obtained at the highest applied force is the ultimate tensile strength. The stiffness is the resistance of material to deformation while Young’s modulus is found by dividing the stress by the strain over the linear portion of stress strain curve [22].

Fifteen dumbbell-shaped tensile specimens were made of UHMWPE—five were soaked in TXA solution (1 g/50 ml saline) for 48 h, five were soaked in 50 ml of saline and the other five were used to standardise the tensile testing machine. The duration of soaking of the tested material was based on the pharmacokinetics properties of TXA in the human body. TXA half-life time is 3.5 h and the human body needs 24 h to clear 90 % of administered TXA.

**Fig. 4:**
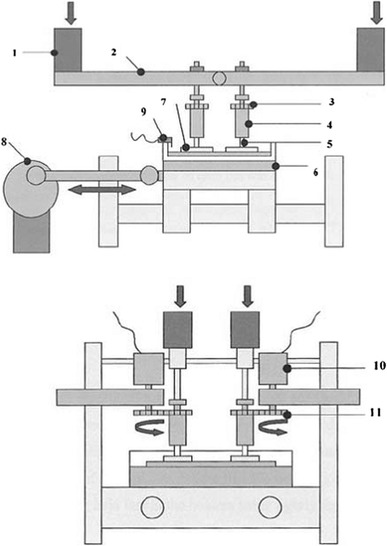
Schematic diagram of the pin-on-plate machine. *1* weight to provide load, *2* lever arm, *3* gear, *4* pin holder, *5* UHMWPE pin, *6* heater bed, *7* Co–Cr–Mo plate, *8* motor to provide reciprocation, *9* level sensor, *10* motor, *11* gear). Reproduced with permission of SAGE Publications Ltd, London, UK

*Wear rate* testing was performed to assess the influence of TXA under accelerated body wear conditions. This test was performed using a multidirectional pin-on-plate machine (Fig. 4). It is a four-station machine applying both reciprocational and rotational motion. The reciprocation was applied by a sledge moving forward and backward over a 4 cm range at 60 cycles/min. The heated bed, lubricant tray, level sensor and plate holders were positioned on this sledge.

The plates were made of Co–Cr–Mo. Four plates were fitted to the tray and secured with three metal plates and screws. They were covered with lubricant and heated to 37° C (body temperature). The pins were made of UHMWPE. Each pin was numbered, notched and tightly fitted to the pin holders at the end of each motor. The rotational frequency was also set at 60 cycles/min (1 Hz).

The lubricant used in all tests was a 24.5 % concentration of bovine serum (protein content 15 g/l) and was prepared following a standardised protocol.

A lubricant level sensor was attached to the lubricant tray to allow the lubricant to be maintained at an almost constant level. Any lubricant loss was assumed to be evaporation of water so this was topped up from a reservoir of distilled water. The rotational motion was provided by four small motors. The four loaded pins were held in stainless steel holders and mounted so that each pin rested on the corresponding plate. A load of 40 N was applied to each station via a static mass and lever arm mechanism.

The wear was assessed gravimetrically (loss of weight) and volumetric loss was calculated by dividing the mass by the density. Twice a week (approximately every 0.25 million cycles), the machine was stopped to allow for gravimetric assessment and machine maintenance. The pins and plates were cleaned¸ dried and weighed following a standardised protocol.

Control specimens were used to take account of the absorption of lubricants by both the pins and plates during the test. The wear volumes were plotted against the sliding distance and the gradient of the line provided the wear rate. Wear rate was divided by the load to determine the wear factor, *k* (mm^3^/Nm).

**Fig. 5:**
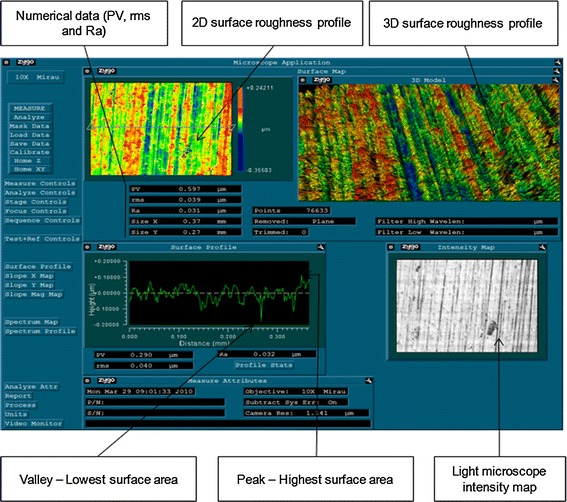
Profilemeter view of surface topography of a Co–Cr–Mo plate

Surface topography measurements were performed using a Zygo NewView 100 non-contacting three-dimensional (3-D) profilemeter. Ten measurements were taken of the pins and plates before and after wear testing. Each measurement provided visual and numerical data of the surface profile of the specimens (Fig. 5). Visual data includes intensity map, 2- and 3-D profiles of surface roughness. This provided a quick scan of a wider area of the specimens to get an overall impression of the roughness of the surfaces. Numerical data included PV, rms and *R*_*a*_.

## Results

The tensile test showed that the stiffness, Young’s modulus, load to fracture and stress at fracture values were not affected by immersion in TXA or saline for 48 h.

The two groups were comparable in term of stiffness, Young’s modulus of elasticity, load at break and stress at break (Table 1). There were no statistically significant differences between saline- and TXA-immersed UHMWPE.

**Table 1:** Tensile test results of the UHMWPE specimens

Variable	Specimens	*N*	Mean (SD)	Mean difference	*P* value (95 % CI)
Stiffness (N/m)	Saline	5	81,588 (10,518)	1,964	0.740 (−15,146 to 11,218)
	TXA	5	83,552 (7,262)		
Young’s modulus (MPa)	Saline	5	923 (119)	23	0.740 (−171 to 127)
	TXA	5	946 (82)		
Load at break (*N*)	Saline	5	330 (33)	12	0.523 (17 to −52)
	TXA	5	342 (20)		
Stress at break (MPa)	Saline	5	47 (5)	1	0.526 (2 to −7)
	TXA	5	48 (3)		

The stiffness, Young’s modulus, load to fracture and stress at fracture of the soaked specimens (TXA and saline) were comparable to those of control specimens (non-soaked specimen) (ANOVA; *P* = 0.79, 0.79, 0.67 and 0.67, respectively).

**Fig. 6:**
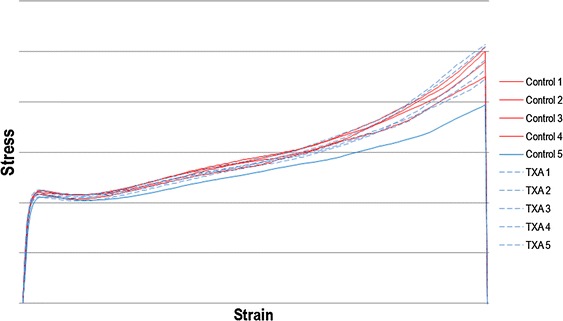
Stress strain curves of tensile. Control specimen number 5 which failed at a lower stress is represented with a *blue solid line* (colour figure online)

The stress strain curves of the ten tensile specimens are shown in Fig. 6. The graph shows all specimens have an almost identical stress strain curve apart from control specimen number five which failed at a lower stress. However, this did not adversely affect the overall findings. It is not unusual to have a faulty specimen with a small scratch or defect that affects its tensile properties.

The wear test involved two multidirectional pin-on-plate machines each with four test stations, four control pins and four control plates which were soaked with saline, and four TXA-soaked pins and four-TXA soaked plates. Another two pins and two plates were used as controls to measure the weight changes caused by lubricant absorption. This was deducted from the test specimens at every gravimetric assessment.

**Fig. 7:**
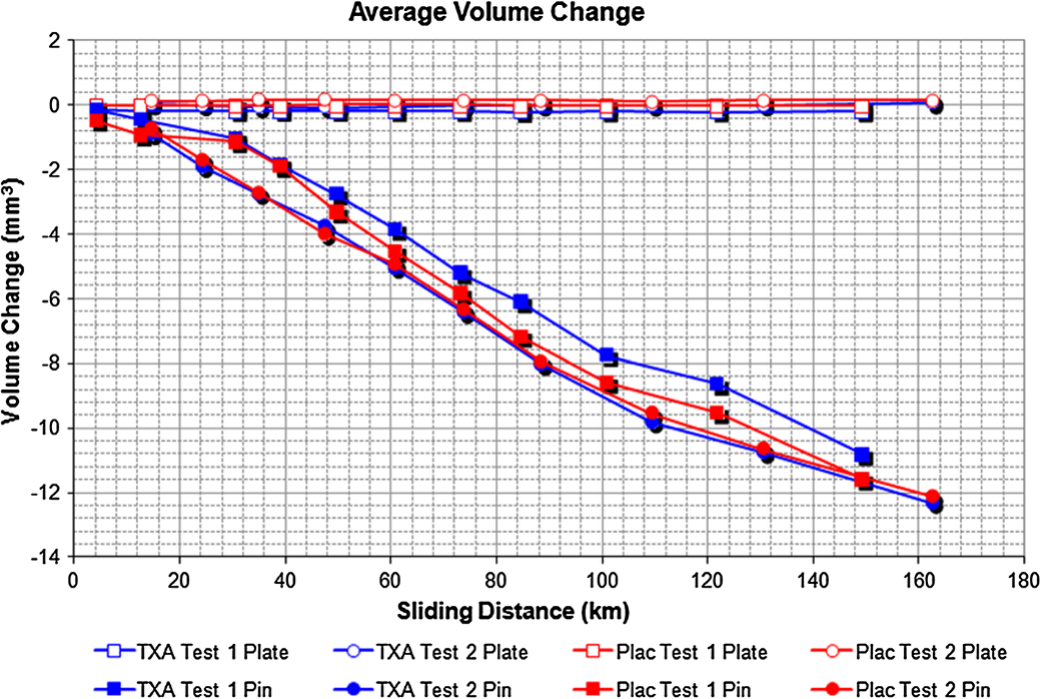
Graph of volume losses of the pins and plates plotted against sliding distance

Table 2 and Fig. 7 show the cumulative volume changes in the pins and plates in each group (placebo or TXA) as measured each time the machines were stopped. As expected, the main weight loss occurred in the UHMWPE pins rather than the Co–Cr–Mo plates as they are softer and prone to wear more rapidly than plates. Wear factors were calculated for all pins and plates using the following equation:

**Table 2:** Cumulative volume loss in mm^3^ in plates and pins

Distance (km)		Plates				Pins			
Test 1	Test 2	TXA test 1 plate	TXA test 2 plate	Placebo test 1 plate	Placebo test 2 plate	TXA test 1 pin	TXA test 2 pin	Placebo test 1 pin	Placebo test 2 pin
0	0	0.00000	0.00000	0.00000	0.00000	0.00000	0.00000	0.00000	0.00000
4	14.2	−0.12659	0.02191	−0.00913	0.13309	−0.14354	−0.87560	−0.46651	−0.71412
12.4	24.2	−0.19679	−0.00406	−0.00162	0.11503	−0.44677	−1.89833	−0.93122	−1.67405
30.5	34.8	−0.19517	−0.07608	−0.00446	0.16575	−1.05862	−2.73446	−1.13756	−2.70755
38.9	47.4	−0.18441	−0.07993	0.00426	0.15276	−1.84989	−3.74284	−1.90192	−3.99762
49.6	60.7	−0.18949	−0.04098	0.01603	0.14566	−2.76676	−5.03112	−3.31760	−4.91449
60.8	73.7	−0.19922	−0.01440	0.00751	0.13511	−3.86485	−6.43065	−4.53590	−6.34094
73.1	88.5	−0.19598	−0.01481	0.01298	0.14262	−5.21055	−8.03652	−5.83495	−7.95219
84.6	109.5	−0.22113	−0.02090	−0.01116	0.09170	−6.11306	−9.82719	−7.16450	−9.54908
100.9	130.6	−0.20308	−0.01075	−0.00122	0.14992	−7.75661	−10.77636	−8.57839	−10.65973
121.9	162.6	−0.21119	0.05336	0.00467	0.14161	−8.64477	−12.32661	−9.52037	−12.13821
149.2		−0.20308		0.00426		−10.81583		−11.57660

$$\mathrm{Wear}\mathrm{factor}=\mathrm{Volume}\mathrm{loss}\left(mm^{3}\right)/\left[\mathrm{sliding}\mathrm{distance}(m)\times \mathrm{load}(N)\right]$$

There were no statistically significant differences between the mean wear factor when comparing either plates or pins which were soaked in saline or TXA as shown in Table 3.

**Table 3:** The wear factors of plates and pins; parametric *t* test

	Groups	*N*	Mean^a^	SD^a^	Mean difference^a^	*P* value (95 % CI)
Plates wear factor	Placebo	4	0.00100	0.013515	0.0035	0.768 (−0.161 to 0.023)
	TXA	4	−0.00250	0.008583		
Pins wear factor	Placebo	4	2.15925	0.240433	0.046	0.677 (−0.319 to 0.411)
	TXA	4	2.11325	0.177184		

^a^×10^−9^ mm^3^/Nm

The surface topography testing revealed no significant difference in the PV, rms and *R*_*a*_ of the plates and pins between the two groups before and after the pin-on-plate wear tests (see Table 4). Hence, the null hypothesis of no difference was supported.

**Table 4:** Post wear surface topography findings

	Group	*N*	Mean	SD	Mean difference	*P* value (95 % CI)
Plates (PV)	Placebo	3	0.279	0.228	−0.0340	0.856 (−0.5 to 0.4)
	TXA	4	0.313	0.240		
Plates (rms)	Placebo	3	0.021	0.0075	−0.0115	0.141 (−0.0284 to 0.0054)
	TXA	4	0.0325	0.0092		
Plates (*R*_*a*_)	Placebo	3	0.019	0.002	−0.007	0.187 (−0.02 to 0.005)
	TXA	4	0.026	0.007		
Pins (PV)	Placebo	4	3.504	1.780	−1.959	0.195 (−5.24 to 1.32)
	TXA	4	5.463	2.010		
Pins (rms)	Placebo	4	0.3167	0.199	−0.0715	0.612 (−0.542 to 0.3531)
	TXA	4	0.3882	0.2216		
Pins (*R*_*a*_)	Placebo	4	0.247	0.119	0.043	0.616 (−0.16 to 0.24)
	TXA	4	0.203	0.113		

*PV* Peak-valley ratio (µm), *rms* root mean square roughness (µm), *R*_*a*_ surface roughness average (µm)

## Discussion

It may take many years to determine the effectiveness of new and innovative designs of artificial joint replacements since many journals refuse to publish new implants series or interventions with <10 years follow-up. Surgeons are cautious about introducing a new intervention without it being carefully studied and assessed. The history of artificial joint replacement supports this caution.

There has been a recent increase in the use of TXA to reduce bleeding and blood transfusion after joint replacement and, more recently, topical application has been advocated without proper safety profile assessment. In this study we investigated the effects of TXA on three important material properties. Tensile testing and surface topography were studied using gold standard methods. This showed there was no difference in the tensile properties and surface topography between the artificial implants that had been soaked in saline and those soaked with TXA.

There are several methods to assess the rate of wear. Clinical and radiological survival analysis of implanted joints is probably the most useful method; however, it takes an extremely long time as some implants can last >30 years. Furthermore, patients are often lost to follow-up during such a long period. Joint simulation is a reliable predictor but it is expensive with limited access. Multidirectional pin-on-plate machines are a reasonable alternative and several studies have confirmed that they are comparable to joint simulators [23–26].

Comparing results from eight stations, there was no significant difference in the wear factor between the two groups and findings were comparable with the wear factors reported in other studies [23, 27].

Authors acknowledge the fact that biomechanical studies do not always correlate well with the clinical outcomes; however, biomechanical tests are widely accepted as the best available predictors and surrogates for future outcomes. Public expectation is that materials used for health reasons need to be tested mechanically or in animal studies before human use. With increasing medical litigation against health care providers, waiting for clinical outcome studies which may take years may not be the option. This study supports the localised usage of TXA around artificial joints used in humans, while accepting the limitations of a laboratory-based study. Separately, members of the team are investigating the 5-year clinical and radiological outcomes of the first 157 patient who underwent total knee replacement using topical TXA in our centre and this will be reported separately.

In summary, laboratory biomechanical testing shows that there are no biomechanical adverse affects on common artificial joint materials exposed to topical TXA. Nonetheless, surgeons using topical TXA are strongly recommended to be vigilant and encouraged to report unexpected premature joint failures associated with topical use of TXA. The National Joint Registry should collect and analyse data on the use of topical TXA, to help inform on-going research into the safety profile of topical TXA in joint replacement.
